# Predicting classes of β-hydroxybutyrate concentrations in blood of dairy cows from milk mid-infrared spectra: Application of a weighted discriminant model addressing class imbalance

**DOI:** 10.3168/jdsc.2026-1074

**Published:** 2026-07-06

**Authors:** M. Lesnoff, C. Grelet, V. Wolf, L. Millot, A. Köck, J. Leblois, B. Bapst, M.A. Crowe, T. Larsen, D.C. Wathes, C.P. Ferris, K.L. Ingvartsen, C. Marchitelli, F. Becker, J. Hummel, M. Gelé, H. Soyeurt, N. Gengler, F. Dehareng

**Affiliations:** 1 SELMET, Université Montpellier, CIRAD, INRAE, Institut Agro, Montpellier, France, 34398; 2 CIRAD, UMR SELMET, Montpellier, France, 34398; 3 ChemHouse Research Group, Montpellier, France, 34398; 4 Walloon Agricultural Research Center (CRA-W), Gembloux, Belgium, 5030; 5 CEL25-90, Roulans, France, 25640; 6 ZuchtData, Vienna, Austria, A-1200; 7 Eleveo, Awé group, Ciney, Belgium, 5590; 8 EEIG European Milk Recording (EMR), Ciney, Belgium, 5590; 9 Qualitas AG, Zug, Switzerland, 6300; 10 University College Dublin (UCD), Dublin, Ireland, D04 C1P1; 11 Department of Animal and Veterinary Sciences, Aarhus University, Tjele, Denmark, DK-8830; 12 Royal Veterinary College (RVC), London, United Kingdom, CM24 1RW; 13 Agri-Food and Biosciences Institute (AFBI), Belfast, Northern Ireland, BT9 5PX; 14 Research Center for Animal Production and Aquaculture (CREA), Rome, Italy, 00184; 15 Institute for Farm Animal Biology (FBN), Dummerstorf, Germany, 18196; 16 University of Göttingen, Göttingen, Germany, D-37075; 17 Idele, Paris, France, 75012; 18 TERRA Teaching and Research Centre, Gembloux Agro-Bio Tech, University of Liège, Gembloux, Belgium, 5030

## Abstract

Monitoring biomarkers in milk or blood is widely used to assess dairy cow health and to detect diseases such as (sub)clinical ketosis. Blood BHB is a key indicator of metabolic imbalance, and its prediction from milk Fourier-transform mid-infrared (FT-MIR) spectra has been extensively explored. However, most of the predictive models applied in spectrometry fail to achieve acceptable accuracy because of the highly skewed distribution of BHB concentrations (many blood samples have low concentrations, but a few have high concentrations). Imbalanced distributions complicate both quantitative and class modeling. Thus, the objective of this work was to undertake discrete predictions of BHB (low versus high level) by implementing a specific weighting in a usual discrimination algorithm to overcome the class imbalance between healthy cows and those suffering from hyperketonemia. The threshold of 1.2 mmol/L was used to distinguish low from high BHB, that is, absence or presence of hyperketonemia. The cleaned dataset combined 4,221 records comprising milk FT-MIR spectra and blood BHB content that were collected across 10 countries between 2013 and 2024, and in multiple breeds, and numerous research projects. Milk MIR spectra (selected wavenumber regions, second derivative Savitzky–Golay preprocessing) served as predictors. A replicated external herd validation strategy was implemented. The weighted model outperformed the standard model, achieving average intraclass error rates of around 18% on external test sets. It reached 82% accuracy, 83% sensitivity, and 81% specificity, with these results comparable to or better than previously published discriminant models, despite using only raw MIR spectra. These findings demonstrate that the use of weighting within discriminant methods is a valid option for the class prediction of asymmetrically distributed health biomarkers.

## Graphical Abstract

**Figure:**
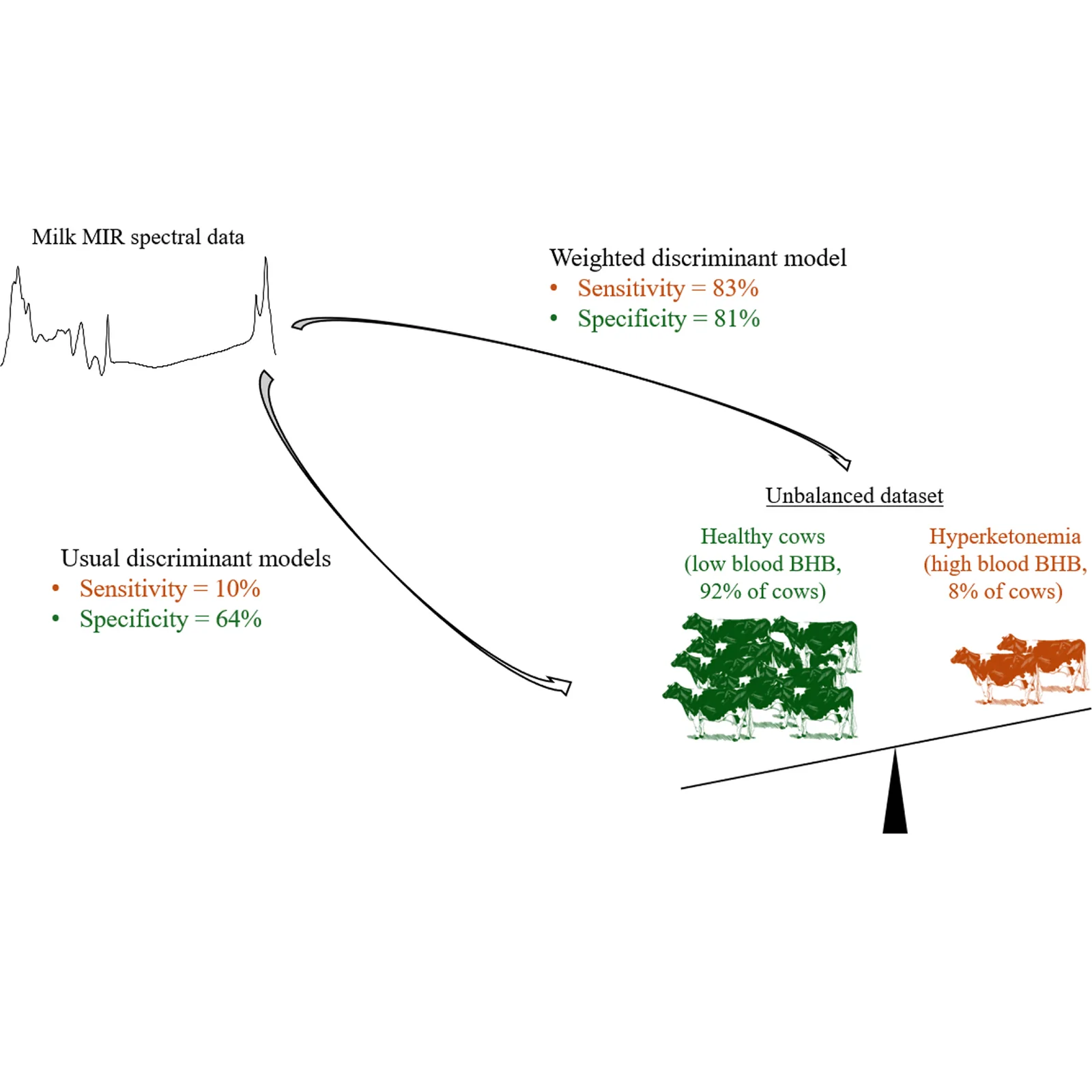

Measuring biomarkers in milk or in blood to provide information on disease status of dairy cows is a common practice, both for routine herd management and genetic studies. Among others, the concentration of blood BHB is routinely used as an indicator of physiological imbalance and hyperketonemia (Moyes et al., 2013; Suthar et al., 2013; McArt et al., 2015). For rapid, large-scale and cost-effective screening of BHB, its prediction from Fourier-transform mid-infrared (**FT-MIR**) spectrum of milk samples has been widely studied. Most previous studies have attempted to predict BHB concentrations using partial least square (**PLS**) regression models (Belay et al., 2017; Benedet et al., 2019a; Grelet et al., 2019; Luke et al., 2019; Walleser et al., 2021; Kostensalo et al., 2023), with the external validation coefficients of determination of these models ranging from 0.21 to 0.70. Other quantitative predictive algorithms, such as elastic net, random forest, gradient boosting, artificial neural networks, and stacking ensembles have also been used, with comparable predictive performances (Pralle et al., 2018; Walleser et al., 2021; Giannuzzi et al., 2023). However, an alternative approach is to seek to identify whether a cow has either a low or a high level of BHB because this discriminates between absence versus presence of hyperketonemia. Therefore, transforming the quantitative concentrations of biomarkers into discrete variables that separate cows with low and high concentrations through a threshold, and using predictive discrimination algorithm, would be very useful (Luke et al., 2019).

Irrespective of methodology adopted, previous authors have highlighted difficulties in modeling blood biomarkers concentrations such as BHB and nonesterified fatty acids (**NEFA**) because of their highly right-skewed distributions (Aernouts et al., 2020; Kostensalo et al., 2023). Indeed, in practice, the majority of samples have a low BHB concentration (from healthy cows) and a few samples from (sub)clinical ketotic cows show an exponential increase of BHB. The resulting right-skewed distribution of the concentrations is particularly difficult to model from spectral data because it goes beyond the linear relationship of milk molecules and spectral absorbance values. When transforming BHB in a discrete variable by using a threshold (i.e., “low” vs. “high” BHB), the issue remains and is translated in a class imbalance, that is, between numbers of healthy cows versus cows in hyperketonemia. Moreover, this issue is observed with many other disease biomarkers, including lactoferrin (Soyeurt et al., 2020) and N-acetyl-β-d-glucosaminidase activity (Grelet et al., 2024) for mastitis, and NEFA as an indicator of negative energy balance (Aernouts et al., 2020). In these scenarios with imbalanced data, modeling methodologies must be adapted (Soyeurt et al., 2020; Kostensalo et al., 2023).

Thus, the objective of this work was to undertake discrete predictions of BHB (low vs. high level) by implementing a specific weighting in a usual discrimination algorithm to overcome the class imbalance between healthy cows and those suffering of hyperketonemia.

Data used in this study (a total of 6,059 milk and blood samples) were collected within 2 international and 7 national projects conducted between 2013 to 2024. Data were collected from 1,799 cows across 83 herds in 10 countries. The dataset involved several breeds (Holstein, Braunvieh, Fleckvieh, Simmental, Brown Swiss, Montbéliarde, Abondance, and crossbred cows), parity ranged from 1 to 13, and cows were between 5 and 226 DIM.

A total of 780 samples were collected within the GplusE project (EU FP7-Project) from AFBI (Agri-Food and Biosciences Institute, Belfast, Northern Ireland), Aarhus University (Tjele, Denmark), CREA (Research Center for Animal Production and Aquaculture, Rome, Italy), CRA-W (Walloon Agricultural Research Centre, Gembloux, Belgium), FBN (Leibniz Institute for Farm Animal Biology, Dummerstorf, Germany) and UCD (University College Dublin, Dublin, Ireland) and in 4 commercial farms in the United Kingdom. A total of 953 samples were collected within the OptiMIR project (EU, Interreg NWE) from 3 experimental farms (Poisy and les Trinottieres, France; Neumuhle, Germany). In national projects, 420 samples were collected from 23 commercial farms in France (within project CEL25-90), 1,017 samples were collected from 49 commercial farms in Austria by Zuchtdata (D4Dairy, FFG comet, project 872039, https://d4dairy.com/), 812 samples from 10 commercial farms involved in 2 Belgian projects (Fertiwalim and Scorewelcow), 345 samples from 10 commercial farms in German IndiKuh project (funding code: 2817905815), and 1,732 samples were collected in Switzerland by Qualitas AG as described in Caldeira et al. (2020).

All milk samples were collected following the guidelines provided by the International Committee for Animal Recording (ICAR Dairy Cattle Milk Recording Working Group, 2023) using ICAR-approved milk samplers. Samples were collected according to either AT or A4 milk recording protocols, namely morning or evening samples, or morning and evening samples pooled into a daily sample with a similar quantity of morning and evening milk as per normal milk recording routines (i.e., 50% from morning and 50% from evening). All milk samples were stored at 4°C until analysis, with 0.02% to 0.03% bronopol used as a preservative. Analyses were undertaken in local laboratories using a wide range of instruments with a total of 36 spectrometers used to analyze the samples: 35 Foss instruments (models FT2, FT6000, FT+, and FT7; Foss, Hillerød, Denmark) and one Standard Lactoscope FT-MIR automatic (Perkin Elmer, previously Delta Instruments, Drachten, the Netherlands). All the spectra from the different instruments were standardized to be merged into a common dataset following the procedure described by Grelet et al. (2015).

Blood samples were collected in the morning (within 3 h of milking) from the tail vein (coccygeal vein). Blood BHB concentration from the coccygeal vein was reported to be similar with jugular vein concentration (Alvarenga et al., 2019). Blood samples within the GplusE, OptiMIR, IndiKuh, and Qualitas AG projects (3,810 samples) were collected in heparin-coated tubes, centrifuged to harvest plasma, and stored at −20°C, with plasma subsequently analyzed by chemical reference methods (e.g., GplusE samples were analyzed at Aarhus University and BHB was determined by measuring absorbance at 340 nm because of the production of NADH at alkaline pH in the presence of BHB dehydrogenase). Samples from CEL25-90, D4Dairy, and the Belgian projects (2,249 samples) were analyzed for BHB using the capillary strip tests WellionVet Belua and Freestyle Optium β-Ketone.

From the 6,059 records, only those with an interval of less than 2 d between milk and blood sampling were retained (693 samples removed). Furthermore, a total of 459 samples were identified as having laboratory analytical issues and were removed. To avoid erroneous associations between spectra and blood samples, as well as analytical issues during analysis, a homemade fat model was applied to the spectra and the generated predictions were compared with the predictions provided by the laboratories. Records with a difference greater than 0.3 g/100 mL (Zhang et al., 2021) between local and laboratory predictions were discarded (47 samples removed). Finally, records from 4 experiments were excluded as a result of errors in sampling, storage, or analytical steps, and the final study dataset consisted of *n*_tot_ = 4,221 samples in 64 herds. The MIR spectra (data **X**) were used as predictors. The spectral areas were selected from ranges (968.1–1,577.5 cm^−1^, 1,731.8–1,762.6 cm^−1^, 1,791.9–1,808.9 cm^−1^, and 2,831.0–2,966.0 cm^−1^) that excluded areas not reproducible between instruments. The spectra were preprocessed by a Savitzky–Golay filter (Luo et al., 2005) with a filter size of 9, a smoothing polynomial order of 3, and a second derivative (Figure 1).

**Figure 1:**
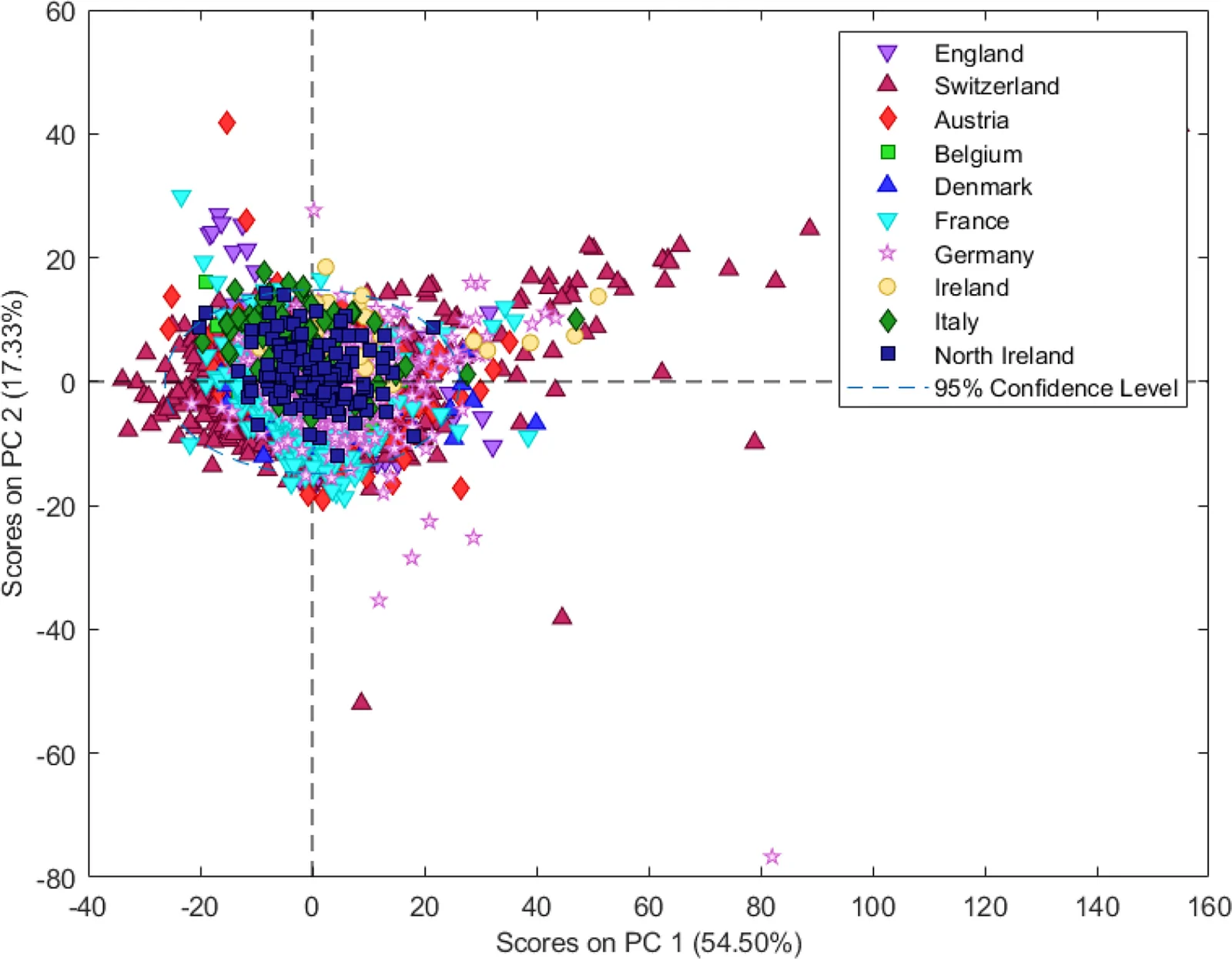
Principal component analysis on the milk MIR spectra (n = 4,221). Spectra were plotted after standardization, first derivative, selection of 212 repeatable wavenumbers and mean-centering and scaling of each wavenumber. PC = principal component.

The response variable to predict (data **y**) was the BHB discrete level defined by 2 classes: “low” (concentration ≤ 1.2 mmol/L), and “high” (concentration >1.2 mmol/L). Cows classified as the high level are considered to suffer from hyperketonemia. Cows were not clinically inspected for presence of ketosis or other diseases, therefore the classification only targets the absence versus the presence of hyperketonemia. The dataset was highly imbalanced in favor of class low, with only 8% of the samples classified as high*.*

The prediction algorithm used as a comparison basis was the simplest partial least squares discriminant analysis (**PLSDA**), often referred as the “usual” or “normal” PLSDA as defined by Brereton and Lloyd (2014). It consists of first transforming the univariate class membership variable to predict to a dummy table (**Y**_dummy_; that is, if there are C classes, a table with C columns of 0/1 binary variables representing the absence/occurrence of each class). Then a PLS regression between the spectra and this dummy table is made: a multivariate PLS is run on data (**X**, **Y**_dummy_) to compute the PLS-score matrix (**T**) and a multiple linear regression model is run on data {**T**, **Y**_dummy_}, giving predictions ${\hat{Y}}_{dummy}.$ For each observation, the final predicted class is the one corresponding to the column that shows the highest value in the predicted dummy table. The usual PLSDA has the advantage of being fast and quite efficient when the number of classes to discriminate is not too large (≤3–4 classes). Nevertheless, it is highly sensitive to class imbalance and generates biased predictions in such cases: The algorithm favors systematically the prediction of the classes that are predominant in the data (Brereton and Lloyd, 2014). A simple approach to remove this bias consists in internally weighting the PLSDA (**WPLSDA**) to give the same importance to each class. Noting *n* is the number of the samples in the training set, the principle in WPLSDA is to embed a vector of weights **w** = (*w*_1_,  . . .*w_n_*) in the PLSDA equations. A detailed description of the general WPLS algorithm used in the study is given in Lesnoff et al. (2020). To control imbalanced class data, vector **w** can be defined such that the sums of the weights by class are equal. In the present case of 2 classes, and noting *p_low_* and *p_high_* the sum of the weights *w_i_* of the samples in class “low” and “high,” respectively, vector **w** is defined such as *p_low_* = *p_high_* = 1/2.

A double replicated K-fold cross-validation (Filzmoser et al., 2009) was implemented to optimize the PLSDA and WPLSDA models and estimate their predictive classification error rates. The following process was repeated *n*_rep_ = 100 times:

Twenty-one herds (∼33% of the total 64 herds) are randomly sampled and define the test set for the ongoing process. The remaining 43 herds define the training set.
On the training set, a replicated K-Fold cross-validation (**C-V**; *K* = 3 and 50 replications) is implemented, and the optimal numbers of latent variables (**LV**) for PLSDA and WPLSDA are selected based on the lower classification error rate averaged over the 50 replications.
The optimal model is fitted again on the (entire) training set and used to predict the test set. The test predictions are used to compute the predictive classification error rates for the given process: global accuracy (overall percentage of well classified samples), sensitivity (percentage of good classification in actual class high), specificity (percentage of good classification in actual class low), positive predictive value (**PPV**; percentage of actual class high in predictions high) and negative predicted value (**NPV**; percentage of actual class low in predictions low).

The final external predictive performances of the 2 models were assessed based on the distributions of the *n*_rep_ = 100 classification test set predictions. For imbalanced data, the naive global classification error rate (i.e., the overall % of misclassified samples [**ERRP**]) computed on C-V or test predictions is a biased estimate of the model performance because it gives too much importance to the dominant class. The arithmetical mean of the intraclass classification error rate (**M-ERRP**), which equalizes the class importance, is a better choice and was used in the present study.

All the computations were implemented with the chemometric package Jchemo version 0.9.1 (Lesnoff, 2021) in the free Julia language version 1.10.0 (Bezanson et al., 2017).

The BHB values ranged from 0.09 to 5.13 mmol/L, with an average of 0.71 mmol/L, SD of 0.49 mmol/L, and a skewness of 3.81. The dataset was highly imbalanced in favor of class low, with only 8% of the samples classified as class high (concentration >1.2 mmol/L). This prevalence of hyperketonemia can be considered low compared with other studies. Benedet et al. (2019b) in a large review reported hyperketonemia prevalence ranging from 11.2% to 47.2%, across several countries and using similar thresholds (1.2 mmol/L in majority of cases). However, most of those studies focused on early lactation only (e.g., from 7 to 14 DIM in Vanholder et al. (2015), in which the maximum prevalence was reported), but our dataset merged records with a large DIM range, with an average of 54 DIM (SD = 48 DIM).

Cross-validations results on the 100 repeated training sets are presented in Figure 2. As expected for imbalanced data, PLSDA was inefficient, with M-ERRP always higher than 40%. The WPLSDA model showed better performances. The optimal dimensions ranged from 5 to 20 LV (mean = 13 LV) with average M-ERRP of approximately 19% (SD = 1.6%).

**Figure 2:**
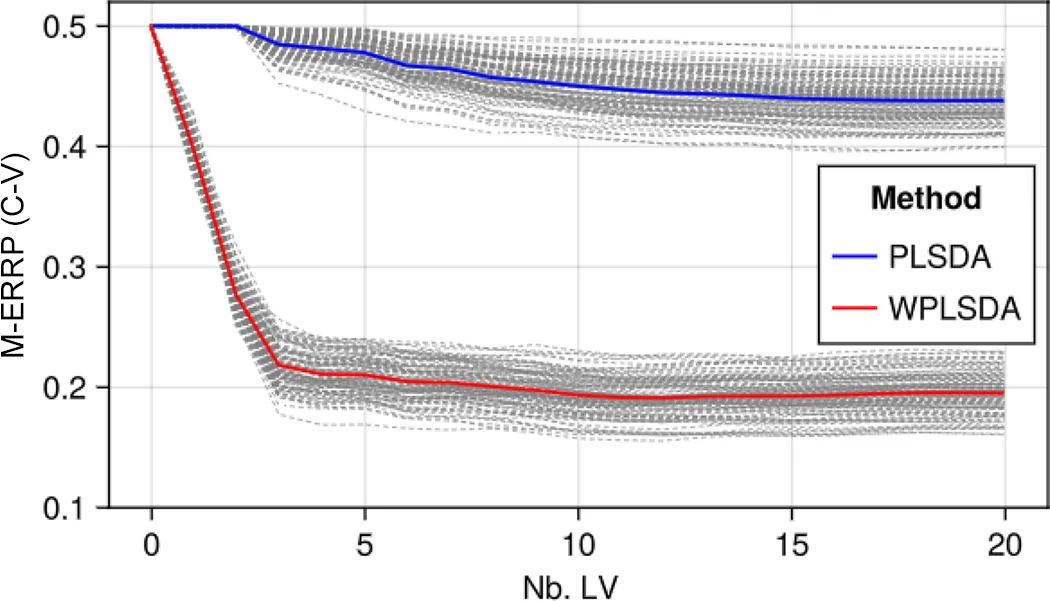
Cross-validation results on the training set for PLSDA and WPLSDA. For each model, the dashed lines represent the 100 repetitions (for each, average of the 50 internal C-V replications), and the red and blue solid lines represent their averages. PLSDA = partial least squares discriminant analysis, WPLSDA = weighted partial least squares discriminant analysis, Nb. LV = number of latent variables.

On the test sets, WPLSDA (for the optimal number of LV) also showed relevant performances, with average M-ERRP of 17.9% (minimum = 11.7%, maximum = 25.4%, SD = 3.9%), with average accuracy of 81.3%, average sensitivity of 83.2%, average specificity of 81.1%, average NPV of 98.2%, and average PPV of 28.0% (Table 1). The bias of the usual PLSDA is clearly apparent in the confusion matrix (Table 1): Predictions were highly favored toward class low independently of the true class, inducing a very low sensitivity (average = 10.2%). The classification performances of WPLSDA can therefore be considered to be good to discriminate both low and high BHB records. However, the approach only partially overcomes the class imbalance issue; the PPV (the real percentage of high values within the total of high predictions) was only 28%, meaning that because of class imbalance, the majority of samples being suspected of hyperketonemia were actually from healthy cows. The profile of the misclassified records should be deeply investigated to see whether these cows were truly negative or close to 1.2 mmol/L threshold and should rather be considered in a gray zone. The effect of this “over-alerting” on the practical use of this tool should also be investigated.

**Table 1:** Confusion matrices for PLSDA and WPLSDA (weighted model) for the blood BHB class predictions from the milk FT-MIR spectra on the external herd validation test sets^1^

Actual true value	PLSDA prediction		Total		WPLSDA prediction		Total	
	Low	High			Low	High		
Low	1,278	6	1,284		1,037	242	1,279	
High	97	11	108		19	94	113	
Total	1,375	17	1,392		1,056	336	1,392	
Specificity, %				99.5				81.1
Sensitivity, %				10.2				83.2
Global accuracy, %				92.6				81.3

1 Each cell of the table represents the average number of samples over the 100 repetitions (averages were rounded to simplify the presentation). PLSDA = partial least squares regression discriminant analysis; WPLSDA = weighted partial least squares regression discriminant analysis.

Only a few studies have used discrimination modeling to predict low versus high BHB concentrations from milk MIR spectra. Gelé et al. (2015) sought to predict the ketosis risk using BHB and NEFA concentrations, obtaining a sensitivity of 81% and a specificity of 69%. Walleser et al. (2023) obtained similar performances with deep learning, gradient boosting machine models, and stacking ensembles. Luke et al. (2019) attempted to model low versus high BHB concentrations, but because of the severe imbalance between groups (high BHB represented only 2% of records) the model was statistically not significant. Pralle et al. (2018) also discriminated low versus high BHB concentrations and obtained a sensitivity of 83% and a specificity of 81%. However, their model included not only the FT-MIR milk spectra but also many other covariables (e.g., milk fatty acids, protein percentage, fat percentage, fat:protein ratio, SCC, urea, milk acetone, milk BHB, calving date, parity, DIM, age at first calving, previous lactation length, previous lactation 305-d mature equivalent milk, gestation length, and dry period length) making their results difficult to compare with the performance of our model. The discrimination with the implemented class-weighting approach proposed in the present study provided similar performance, but using only the FT-MIR spectra. The current results suggest that combining data from multiple sources and countries, as well as using weighted modeling algorithms, is a good option to predict traits with specific distributions, such as BHB. The model was tested in a robust external herd validation, and results are expected to be similar in real field conditions.
